# Supplementary material for: Hybridization History and Repetitive Element Content in the Genome of a Homoploid Hybrid, Yucca gloriosa (Asparagaceae)
Source: Front Plant Sci. 2021 Jan 15;11:573767. doi: 10.3389/fpls.2020.573767 (PMC7843428; doi:10.3389/fpls.2020.573767)
Supplement: Supplementary file 1 [file Data_Sheet_1.PDF]

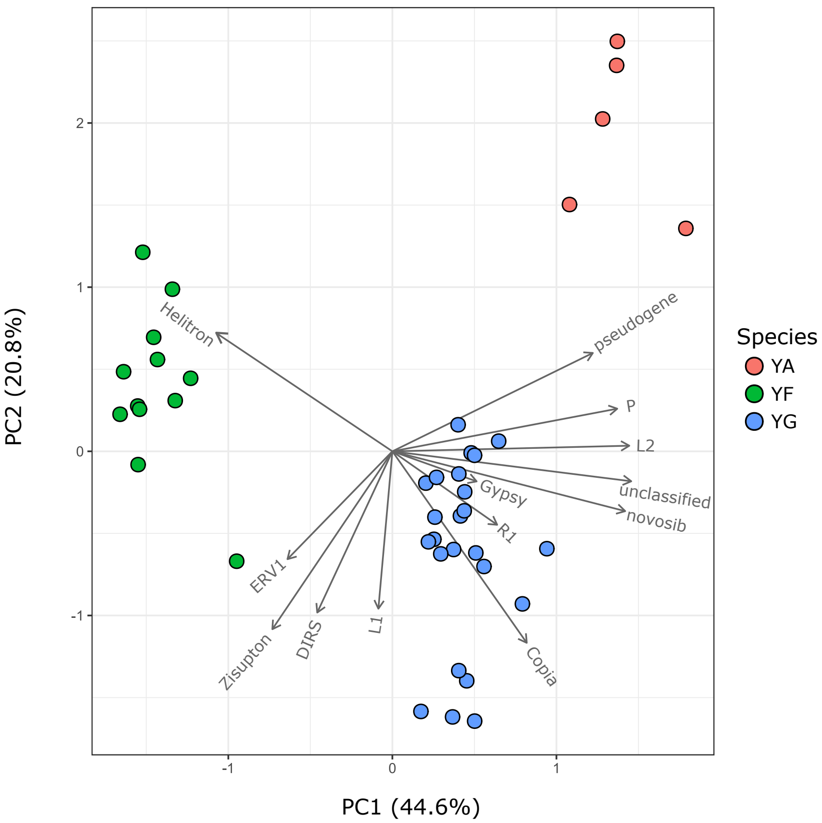

Supplemental Figure 1 - Principal Coordinates Analysis of repeat subfamily abundance in genotypes of three *Yucca* species.
